# Circulating dipeptidyl peptidase-4 is independently associated with the presence and severity of NAFLD/NASH in individuals with and without obesity and metabolic disease

**DOI:** 10.1007/s40618-020-01392-5

**Published:** 2020-08-27

**Authors:** Ilaria Barchetta, Valentina Ceccarelli, Flavia A. Cimini, Eugenio Barone, Federica Sentinelli, Mariagrazia Coluzzi, Caterina Chiappetta, Laura Bertoccini, Antonella Tramutola, Giancarlo Labbadia, Claudio Di Cristofano, Gianfranco Silecchia, Frida Leonetti, Maria G. Cavallo

**Affiliations:** 1 Department of Experimental Medicine, Sapienza University of Rome, Rome, Italy; 2 Department of Biochemical Sciences “A. Rossi-Fanelli”, Sapienza University of Rome, Rome, Italy; 3 Department of Medical-Surgical Sciences and Bio-Technologies, Sapienza University of Rome, Rome, Italy; 4 Department of Internal Medicine and Medical Specialties, Sapienza University of Rome, Rome, Italy

**Keywords:** DPP4, Fatty liver

## Abstract

**Introduction:**

Dipeptidyl peptidase 4 (DPP4) levels are associated to metabolic and cardiovascular diseases in humans; initial evidence reported a relationship between DPP4 and chronic liver diseases. Aim of this study was to investigate hepatic and systemic DPP4 levels/activity in relation to NAFLD/NASH in individuals with and without metabolic disease.

**Methods:**

We recruited fifty-two obese individuals undergoing bariatric surgery and intra-operative liver biopsy at Sapienza University, Rome, Italy. The association between DPP4 levels/activity and NAFLD was also evaluated in 126 non-obese individuals recruited in the same setting.

**Results:**

NAFLD patients had significantly higher circulating DPP4 activity than no-NAFLD in both the obese and non-obese cohorts; plasma DPP4 activity and levels linearly correlated with steatosis grade and inflammation at the liver biopsy. Hepatic *DPP4* mRNA was not associated to either its circulating levels/activity or NAFLD. In the multivariate logistic regression analysis on all the study participants (*n* = 178), higher circulating DPP4 activity was associated with NAFLD independently of potential confounders with OR (95% CI): 3.5 (1.2–10.21), *p* = 0.022.

**Conclusions:**

This study demonstrates the coexistence of increased plasma DPP4 levels and activity in NAFLD. Circulating DPP4 measurement may represent a novel cost-effective strategy for NAFLD/NASH risk stratification and a potential tool for monitoring disease’s progression in established NAFLD.

## Background

Non-alcoholic fatty liver disease (NAFLD) is a chronic pathological condition characterized by an excessive deposition of triglycerides in the hepatocytes and has become one of the most epidemic hepatic diseases worldwide [1]. NAFLD development is tightly connected to the presence of metabolic diseases, so that its incidence has considerably increased in the last decades, paralleling the growing prevalence of obesity and type 2 diabetes mellitus (T2DM) [2].

Along with insulin resistance, multiple additional hits are necessary for NAFLD to develop and eventually to progress to steatohepatitis (NASH), cirrhosis and hepatocarcinoma [3–5]. Accumulating evidences suggest a possible role of the dipeptidyl-peptidase 4 (DPP4) in mediating chronic liver damage in experimental models [6–8]. DPP4 is a serine protease which acts by cleaving a variety of substrates including incretin hormones, cytokines, growth factors and neuropeptides [9, 10]; it is constitutively expressed in a membrane-bound form by several cell types, i.e. T-lymphocytes, endothelial cells, muscle cells, adipocytes and hepatocytes [11] and is released by proteolytic cleavage as a soluble form in the bloodstream [6, 10].

DPP4 is highly expressed in liver [7, 8]; increased hepatic and circulating DPP4 levels have been associated with chronic hepatic diseases in both humans [12–14] and murine models [8, 15]. Transgenic mice with hepatocyte-specific DPP4 overexpression fed with high-fat diet (HFD) displayed greater hepatic fat content and signs of liver damage than wild type [8]; conversely, DPP4 knocking out was shown to protect mice from experimentally-induced liver injury [15].

Therefore, it is plausible to hypothesize that DPP4 may play a role also in human NAFLD and, in consideration of the characteristics of this enzyme, which is cleaved and released in the bloodstream in relation to its tissue expression, its circulating concentration and activity may reflect the severity of liver impairment in presence of NAFLD and NASH.

Studies investigating hepatic or circulating DPP4 levels in relation to the presence of NAFLD in humans, found controversial results [13, 14, 16, 17]; furthermore, no evidence on the contextual expression of *DPP4* mRNA in the liver, along with the measurement of its plasma concentration and activity is available yet.

Therefore, aim of this study was to investigate the relationship between hepatic and systemic DPP4 levels/activity and the presence of NAFLD/NASH in individuals with and without obesity and T2DM.

## Methods

### Population

For this study, we recruited fifty-two consecutive morbid obese individuals undergoing bariatric surgery for clinical indication at Sapienza University of Rome, Italy. The presence of an association between circulating DPP4 and NAFLD/NASH was further explored in an additional cohort of non-obese individuals (*n* = 126) with or without T2DM and metabolic syndrome (MS), referring to the same outpatient clinics for metabolic evaluations, including upper abdomen ultrasonography (US) for assessing the presence of hepatosteatosis.

To be eligible in this study, all participants had to fulfil the following inclusion criteria: male or female subjects aged between 20 and 65 years; no history of excessive alcohol drinking (considered as an average daily consumption of alcohol > 30 g/day in men and > 20 g/day in women); negative tests for the presence of hepatitis B surface antigen and antibody to hepatitis C virus; absence of history of cirrhosis and other causes of liver diseases (hemochromatosis, autoimmune hepatitis, Wilson’s disease); no treatment with drugs known to cause liver steatosis (e.g., corticosteroids, oestrogens, methotrexate, tetracycline, calcium channel blockers and/or amiodarone).

The study protocol has been reviewed and approved by the local Ethics Committee and conducted in conformance with the Helsinki Declaration. All the study participants signed a written informed consent before undergoing all the study procedures.

### Clinical and laboratory measurements

All study participants underwent medical history collection and clinical work-up. Weight and height were measured light clothes on and without shoes and the body mass index (BMI, Kg/m^2^) was calculated. Waist circumference (cm) was measured midway between the 12th rib and the iliac crest. Systemic systolic and diastolic blood pressure (SBP, DBP; mmHg) were measured after five minutes resting; three measurements were taken and the average of the second and third one was recorded and used in the analyses. Fasting venous blood samples were drawn for routine biochemistry and metabolic profiling. Fasting blood glucose (FBG, mg/dL), glycosylated haemoglobin (HbA1c, %—mmol/mol), total cholesterol (mg/dL), high-density lipoprotein cholesterol (HDL, mg/dL), triglycerides (mg/dL), aspartate aminotransferase (AST, IU/L), alanine aminotransferase (ALT, IU/L), gamma-glutamil transpeptidase (GGT, IU/L), creatinine (mg/dL) and conjugated/unconjugated bilirubin (mg/dl) measurement were performed by standard laboratory methods. The FIB-4 score was calculated to estimate liver fibrosis [18]. Low-density lipoprotein (LDL) cholesterol was calculated by the Friedwald formula. Fasting blood insulin (FBI, IU/mL) was measured by radioimmunoassay (ADVIA Insulin Ready Pack 100; Bayer Diagnostics, Milan, Italy; intra- and inter-assay coefficients of variation < 5%).

Circulating DPP4 activity was assessed according to Matheeussen et al. [19] by measuring the 7-amino-4- methylcoumarin (AMC) cleavage rate from the synthetic substrate H-glycyl-prolyl-AMC (H-Gly-Pro-AMC; BioVision, San Francisco, California, USA, nmol/min/ml) on plasma samples frozen immediately after separation and stored at − 25 °C for few weeks. To attribute the Gly-Pro liberating activity solely to DPP4 and not to other members of DPP4 family, a parallel set of samples was incubated with the selective DPP4 inhibitor, sitagliptin, at a final concentration of 10 mM. For each sample, DPP4 activity was then calculated based on the residual fluorescence obtained by subtracting the fluorescence of inhibited sample from those of non-inhibited sample.

Plasma DPP-4 concentration was assessed by sandwich ELISA (R&D Systems, Minneapolis, MN), according to the manufacturer’s instructions.

MS was diagnosed according to the modified NCEP ATP-III criteria [20]. Diabetes mellitus was defined according to the American Diabetes Association 2020 criteria [21].

### Liver histology and gene expression analyses

Morbid obese patients (*n* = 52) underwent intra-operative liver biopsies during sleeve-gastrectomy intervention, following the recommendations by the American Association for the Study of Liver Diseases [22]. A fragment length of 15 mm or the presence of 10 complete portal tracts was required for clinical diagnosis [23]. A single pathologist blinded to patients’ medical history and biochemistry performed clinical histology and immunohistochemestry. Liver fragments were fixed in buffered formalin for 2–4 h and embedded in paraffin, sections were cut and stained with haematoxylin and eosin and Masson’s trichrome stains. NAFLD was classified based on Brunt score [24], the presence of NASH was detected by the contextual detection of steatosis, ballooning and lobular inflammation; NAFLD activity score (NAS) was calculated as the sum of scores for steatosis, lobular inflammation, and ballooning [25]; fibrosis was quantified on the basis of the NASH Clinical Research Network Scoring System Definition [25].

PCR product of human *DPP4* was detected by using gene-specific primers and probes labelled with reporter dye FAM (gene ID: 1803, Applied Biosystems, Foster City, CA, USA). GAPDH was used as an internal standard which yielded a predicted amplicon of 171 bp. GAPDH was detected using gene-specific primers and probes labelled with reporter day FAM (gene ID: 2597, Applied Biosystems, Foster City, CA, USA).TaqMan real-time quantitative PCR was performed on an ABI PRISM 7500 Fast Real-Time PCR System (Applied Biosystem, Foster City, CA, USA). PCR reactions were carried out in triplicate on 96-well plates with 10 uL per well using 1X TaqMan Master Mix and the results were evaluated at the end of the reaction using the ABI PRISM 7500 software (Applied Biosystem, Foster City, CA, USA).

### NAFLD assessment in non-obese individuals

In the additional study cohort of individuals without severe obesity, NAFLD was diagnosed by clinical criteria in presence of fatty liver at the upper abdomen US examination (Esaote, Genoa, Italy), which was conducted by the same operator blinded to laboratory values. Liver steatosis was defined on the basis of abnormally intense, high-level echoes arising from the hepatic parenchyma, liver-kidney difference in echo amplitude, echo penetration into the deep portion of the liver and clarity of liver blood vessel structure [26].

### Statistics

For this purposes of our study, the sample size was calculated according to results obtained in a previous research investigating circulating DPP4 activity, set as the primary outcome of our study, in individuals with and without biopsy-proven NASH [16]. Thus, we found that a sample size of 13 individuals for each group (i.e. a total sample size of 26, assuming equal group sizes), would have allowed to achieve a power of 90% and a level of significance of 1% (two sided), for detecting a true difference in means between the test and the reference group [27].

Continuous variables are reported as the mean ± standard deviation (SD) and categorical variables as percentages. Skewed variables underwent natural logarithmic transformation before performing the analyses. Comparisons between two groups were performed by Student's *T* test or Mann–Whitney for continuous variables and $\chi^{2}$ test for categorical parameters, as appropriate. Correlations between continuous variables were calculated by Pearson’s or Spearman’s coefficient. The existence of an independent association between circulating DPP4 activity and NAFLD was tested by multiple logistic regression models adjusted for sex and age and potential clinical determinants of NAFLD. SPSS version 25 (IBM, Armonk, NY) was used to perform all the statistical analyses. Two-sided *p* value < 0.05 was considered statistically significant, with a confidence interval of 95% (95% CI).

## Results

### DPP4 and biopsy-proven NAFLD

Thirty-two out of fifty-two (61%) patients had biopsy-proven NAFLD; characteristics of the study population in relation to the presence of NAFLD are shown in Table 1.

**Table 1:** Characteristics of the study population according to the diagnosis of NAFLD

	No NAFLD (*n* = 20)	NAFLD (*n* = 32)	*p* value^b^
Age (years)	42.13 ± 8.7	45.22 ± 10.9	0.35
Gender (F%)	87%	65%	0.03^a^
BMI (Kg/m^2^)	43.8 ± 6.1	42.1 ± 5.4	0.37
Waist circumference (cm)	128.1 ± 12.5	127.7 ± 16.1	0.94
PAS (mmHg)	129.2 ± 10.3	126.9 ± 11.8	0.47
PAD (mmHg)	84.2 ± 6.9	84.9 ± 17.4	0.86
AST (IU/l)	20.8 ± 7.8	29 ± 13.7	0.032
ALT (IU/l)	21.9 ± 10.4	41 ± 23.2	< 0.001
GGT (UI/l)	20.16 ± 7.2	30.8 ± 17.7	0.009
Total cholesterol (mg/dl)	204.7 ± 30.6	190.6 ± 29.6	0.15
HDL-C (mg/dl)	48.8 ± 11.3	45.4 ± 10.7	0.34
LDL-C (mg/dl)	131.6 ± 23.1	113.6 ± 26.6	0.03
Triglycerides (mg/dl)	109 ± 39	167.1 ± 74.5	0.006
Total bilirubin (mg/dl)	3.9 ± 6.7	2.25 ± 3.9	0.34
Direct bilirubin (mg/dl)	1.40 ± 2.84	0.92 ± 1.9	0.58
FBG (mg/dl)	96.8 ± 12.9	103.8 ± 23	0.23
HbA1c (%)	5.3 ± 0.3	5.7 ± 0.63	0.05
FBI (IU/mL)	11.9 ± 7	15.3 ± 8.2	0.29
HOMA-IR	2.85 ± 1.5	3.82 ± 2.4	0.24
HOMA-β%	149.6 ± 145.7	169.2 ± 70.5	0.69
FIB-4	0.8 ± 0.25	1.04 ± 0.3	0.039
Steatosis % [median(range)]	3.5 (0.75–5)	60 (30–70)	< 0.001
Steatosis grade (*n*, 0/1/2/3)	20/0/0/0	0/8/8/16	< 0.001
Lobular inflammation (*n*, 0/1/2)	8/12/0	14/8/10	0.10
Ballooning (n, 0/1/2)	19/1/0	12/15/5	< 0.001
Fibrosis (F0/F1/F2/F3/F4)	10/10/0/0/0	10/18/3/1/0	0.20

*T* test applied

^a^$$\chi^{2}$$ test applied

^b^Mann–Whitney test applied. Statistical significance is related to the comparison between NAFLD and non-NAFLD sub-groups

Plasma DPP4 activity was significantly higher in NAFLD than in no-NAFLD patients (209,481.8 ± 62,658.3 nmol/min/ml vs. 167,287 ± 61,513.2 nmol/min/ml, *p* = 0.02) and directly correlated with the severity of hepatic impairment, as expressed by greater steatosis grade (*r* = 0.33, *p* = 0.04) and lobular inflammation (*r* = 0.31, *p* = 0.03) at the biopsy and higher AST (*r* = 0.41, *p* = 0.027), ALT (*r* = 0.30 *p* = 0.03), GGT (*r* = 0.41, *p* = 0.017), LDL cholesterol (*r* = 0.31, *p* = 0.048) and FIB-4 (*r* = 0.52, *p* = 0.004).

Correlations between plasma DPP4 activity and clinical parameters are summarized in Table 2.

**Table 2:** Plasma DPP4 activity—bivariate correlation analysis

	Correlation coefficient^b^	*p* value
AST	0.41	0.027
ALT	0.28	0.042
GGT	0.41	0.017
LDL cholesterol	0.31	0.048
FIB-4	0.52	0.004
Steatosis grade	0.33	0.04^a^
Lobular inflammation	0.31	0.03^a^

^a^Spearman’s coefficient

^b^Pearson’s coefficient

The association between higher circulating DPP4 activity and the presence of NAFLD persisted statistically significant, after adjusting for age, sex and T2DM diagnosis (*β* coefficient = 3.19, *p* = 0.025).

NAFLD patients displayed a trend towards higher plasma DPP4 concentration than no-NAFLD, although this difference did not reach the statistical significance (448.5 ± 103.2 ng/ml vs. 341.4 ± 128.4 ng/ml, *p* = 0.06). As observed for circulating DPP4 activity, also plasma DPP4 concentration positively correlated with steatosis grade (*r* = 0.71, *p* = 0.032), lobular inflammation (*r* = 0.5, *p* = 0.024) and ALT levels (*r* = 0.48, *p* = 0.03).

**Fig. 1:**
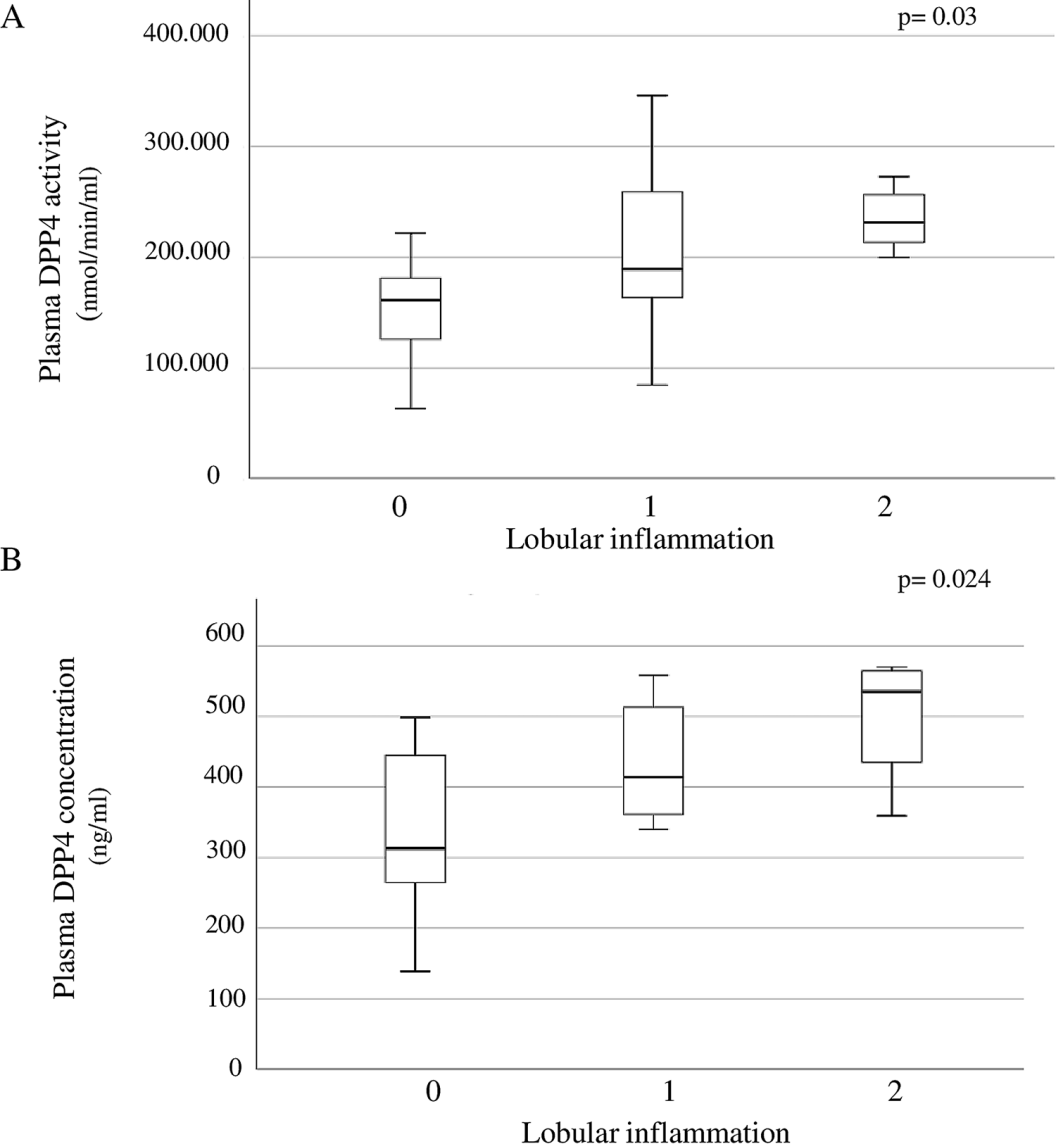
Median (interquartile range) DPP4 activity (**a**) and concentration (**b**) in relation to NAFLD severity, as expressed by presence and grade of lobular inflammation

Figure 1 shows circulating median and interquartile range of DPP4 activity and concentration in relation to NAFLD/NASH severity, as expressed by presence and grade of lobular inflammation.

Hepatic *DPP4* mRNA expression was detected in 85% of study participants; no different hepatic *DPP4* mRNA expression was found between patients with or without NAFLD (0.052 ± 0.79 vs. 0.033 ± 0.032 arbitrary units (AU), *p* = 0.35; Mann–Whitney test applied). Hepatic *DPP4* levels were not associated with either histological/clinical indexes of liver injury or with the presence of metabolic disease, such as MS and T2DM. Liver DPP4 mRNA expression levels did not correlate with plasma DPP4 concentration and activity (not shown).

### DPP4 and US-detected NAFLD

In order to confirm the finding of an association between increased plasma DPP4 activity and NAFLD diagnosis in non-severe obese subjects, we analysed data from an additional cohort of 126 individual with and without MS and/or T2DM undergoing liver US examination to screen NAFLD. Eighty-nine individuals (71%) had US-detected fatty liver, both plasma DPP4 activity and DPP4 concentration were significantly higher in NAFLD in comparison to no-NAFLD individuals (plasma DPP4 activity = NAFLD: 222,822 ± 90,669.3 nmol/min/ml vs. no-NAFLD: 173,059.5 ± 91,040 nmol/min/ml, *p* = 0.006; plasma DPP4 concentration = NAFLD: 533.6 ± 161.9 ng/ml vs. no-NAFLD: 453.7 ± 125.8 ng/ml, *p* = 0.037). Clinical and metabolic characteristics of this study cohort are described in Table 3.

**Table 3:** Characteristics of the study population according to the presence of US-detected NAFLD

	No NAFLD (*n* = 37)	NAFLD (*n* = 89)	*p* value^b^
Age (years)	49 ± 12.1	52.7 ± 9.9	0.021
Gender (%F)	57%	40%	0.013^a^
BMI (Kg/m^2^)	26.4 ± 5.6	30.4 ± 4.9	< 0.001
Waist circumference (cm)	91.4 ± 18.1	103.5 ± 12.8	< 0.001
SBP (mmHg)	119.2 ± 19.2	130.4 ± 15.9	< 0.001
DBP (mmHg)	77.2 ± 8.1	80.6 ± 10	0.019
AST (IU/l)	19.1 ± 5.8	24.8 ± 12.6	< 0.001
ALT (IU/l)	21.1 ± 11	34.9 ± 23.6	< 0.001
GGT (IU/l)	19.8 ± 14.5	42.8 ± 59.5	< 0.001
Total cholesterol (mg/dl)	194.7 ± 38.7	188.2 ± 40.8	0.27
HDL-C (mg/dl)	55.3 ± 12.8	48.8 ± 13.3	< 0.001
LDL-C (mg/dl)	115 ± 38.2	110.6 ± 38.6	0.46
Triglycerides (mg/dl)	106.5 ± 54.7	146.8 ± 81.8	< 0.001
Creatinine (mg/dl)	2.2 ± 11.7	0.86 ± 0.25	0.35
Uric acid (mg/dl)	5 ± 1.4	5.5 ± 1.2	0.12
FBG (mg/dl)	98 ± 27.9	122.8 ± 34.4	< 0.001
HbA1c (%)	8.25 ± 2.6	6.6 ± 0.9	0.052
FBI (IU/mL)	19.1 ± 11.8	25 ± 26.3	0.058
HOMA-IR	4.8 ± 3.2	7.1 ± 7.6	0.018
HOMA-β%	243.5 ± 189.5	182.1 ± 186.6	0.10
MS (%)	25%	70%	< 0.001^a^
T2DM (%)	13%	66%	< 0.001^a^
DPP4 activity (nmol/min/ml)	173,059.5 ± 91,040	222,822 ± 90,669.3	0.006
DPP4 concentration (ng/ml)	453.7 ± 125.8	533.6 ± 161.9	0.055

*T* test applied

^a^$$\chi^{2}$$ test applied

^b^Mann–Whitney test applied. Statistical significance is related to the comparison between NAFLD and non-NAFLD sub-groups

Increased plasma DPP4 activity was associated with higher circulating DPP4 (*r* = 0.55, *p* < 0.001), AST (*r* = 0.22, *p* = 0.017), ALT (*r* = 0.22, *p* = 0.016) and GGT (*r* = 0.27, *p* = 0.004) levels and with the presence of NAFLD (*r* = 0.28, *p* = 0.001), MS (*r* = 0.20, *p* = 0.019) and T2DM (*r* = 0.28, *p* = 0.001) (Table 4).

**Table 4:** Bivariate correlation analyses between (A) plasma DPP4 activity and (B) soluble DPP4 concentration and covariates in the cohort individuals with and without US-assessed NAFLD (*n* = 126)

	A		B	
	DPP4 activity		Soluble DPP4 concentration	
	*r* coefficient	*p* value	*r* coefficient	*p* value
Age	0.13	0.15	0.16	0.14
Sex	0.08	0.37	0.09	0.44
BMI	0.16	0.08	0.23	0.51
Waist circumference	0.15	0.12	0.09	0.48
FBG	0.15	0.10	0.25	0.033
HbA1c	− 0.48	0.71	0.12	0.41
Total cholesterol	0.10	0.28	0.16	0.17
HDL	0.04	0.64	0.12	0.31
LDL	0.08	0.39	0.07	0.53
Triglycerides	0.11	0.21	− 0.005	0.96
AST	0.22	0.017	0.28	0.02
ALT	0.22	0.016	0.25	0.033
GGT	0.27	0.004	0.26	0.029
HOMA-IR	− 0.38	0.74	0.44	0.024
HOMA-β	− 0.11	0.34	0.31	0.13
T2DM	0.28	0.001	0.41	< 0.001
MS	0.20	0.019	0.27	0.015
NAFLD	0.28	0.001	0.24	0.036
FIB4	0.17	0.21	0.07	0.66

Plasma DPP4 activity and concentration are considered as continuous variables; *r* Spearman’s coefficient

Plasma DPP4 levels were associated with higher FBG (*r* = 0.25, *p* = 0.03), AST (*r* = 0.28, *p* = 0.02), ALT (*r* = 0.25, *p* = 0.033), GGT (*r* = 0.26, *p* = 0.029) and HOMA-IR (*r* = 0.44, *p* = 0.024). As observed for circulating DPP4 activity, plasma DPP4 concentration associated with the presence of NAFLD (*r* = 0.24, *p* = 0.03), MS (*r* = 0.27, *p* = 0.015) and with T2DM diagnosis (*r* = 0.41, *p* ≤ 0.001) (Table 4).

Greater DPP4 activity was associated with the presence of NAFLD in a multivariate logistic model adjusted for sex, age, BMI, HOMA-IR and MS (*β* coefficient = 0.52, *p* = 0.009).

Finally, in the whole study population (*n* = 178), higher plasma DPP4 activity independently associated to the diagnosis of NAFLD with an OR: 3.504 (95% CI 1.20–10.21, *p* = 0.022) regardless of potential confounders, as shown in the multivariate logistic regression analysis (Table 5).

**Table 5:** Multivariate logistic regression analysis for NAFLD (yes/no) in the whole study population (*n* = 178)

	*β* coefficient	Standard error	*p* value	Odds ratio	95% Confidence Interval	
Constant	− 1.602	4.241	0.71	0.20	–	–
DDP4 activity	1.254	0.545	0.02	3.504	1.203	10.205
BMI	− 0.006	01.31	0.96	0.994	0.769	1.284
Age	− 0.008	0.039	0.847	0.993	0.919	1.072
Sex (M/F)	− 1.372	0.997	0.169	0.254	0.036	1.788
Triglycerides	0.005	0.010	0.651	1.005	0.985	1.025
MS (yes/no)	2.986	1.521	0.050	19.81	1.0004	390.7
HOMA-IR	− 0.05	0.110	0.642	0.950	0.767	1.178

## Discussion

The main finding of this study is that individuals with NAFLD have increased plasma DPP4 activity than no-NAFLD subjects, regardless of the presence of obesity and metabolic disease.

In NAFLD patients, both plasma DPP4 activity and concentration linearly correlated with hepatic inflammation and steatosis grade at the liver biopsy, and with clinical markers of hepatic damage. Differently, no relationship was found between hepatic *DPP4* mRNA expression and NAFLD. Moreover, liver *DPP4* mRNA was not associated to either circulating DPP4 activity or concentration.

This is the first study investigating contextually circulating DPP4 enzyme activity, as well as its plasma concentration and hepatic expression, in relation to the presence of NAFLD.

Some studies reported altered DPP4 levels in a number of hepatic diseases, such as chronic C hepatitis [28], hepatocarcinoma and cirrhosis [7]; however, data in literature on circulating DPP4 activity and its concentration in NAFLD/NASH are contrasting [13, 16, 17, 29]. DPP4 activity was demonstrated to be increased in NAFLD patients and correlated with blood transaminases and systemic inflammatory markers [17, 29]; however, in these studies, no data on plasma DPP4 concentration and liver *DPP4* levels were available [17, 29].

Williams et al. found an association between circulating DPP4 activity and markers of hepatic fibrosis, but no relationship with hepatic fat content [13]. Balaban et al. demonstrated, in a small cohort of individuals, that serum DPP4 activity was significantly higher in NASH patients than in normal-liver subjects and correlated with NASH severity and steatosis grade; however, no association was found between DPP4 activity and liver enzymes or metabolic parameters [16]. Again, in these studies, no data regarding plasma DPP4 concentration and/or hepatic DPP4 expression were available [13, 16].

In our research, both DPP4 activity and concentration were associated to more severe steatosis grade and inflammation at the biopsy whereas hepatic *DPP4* expression was not influenced by liver damage in the course of NAFLD/NASH.

Previously, Baumeier and collaborators demonstrated a correlation between hepatic DPP4 DNA methylation and stages of hepatosteatosis and NASH [12]. Another report showed increased DPP4 mRNA levels in NAFLD vs. no-NAFLD patients; however, in this study, NAFLD individuals had significantly higher BMI than normal-liver participants and, therefore, an influence of total fat mass in the relationship between hepatic *DPP4* and NAFLD could not be ruled out [14].

Another finding of our study is that circulating DPP4 activity and concentration are increased in presence of MS and T2DM. Previous investigations demonstrated altered DPP4 activity/concentration in relation to metabolic diseases such as obesity [30–32] and T2DM [31, 33]. Despite our study confirmed the existence of an association between circulating DPP4 and T2DM, no relationship was found between DPP4 and BMI. Thus, our overall findings point toward a role for DPP4 in identifying NAFLD and metabolic diseases in individuals with and without obesity and T2DM, instead of being associated with fat mass and body adiposity itself.

In our study, DPP4 activity was independently associated with the presence of NAFLD, after adjustment for potential cofounders such as obesity, metabolic disease and insulin resistance. Indeed, our data depict DPP4 as a potential biomarker of hepatic injury in the course of metabolic disturbance.

Many reports attribute to the adipose tissue a role as a major contributor of DPP4 levels into the bloodstream [34, 35]. Thus, the expression and release of DPP4 from sources other than liver, may explain the absence of a significant correlation, in our study, between the hepatic *DPP4* and its circulating concentration.

In our NAFLD/NASH patients, liver *DPP4* levels do not seem to be related to either the hepatic fat content or inflammation and fibrosis. Hepatic *DPP4* has been shown to be increased in severe liver diseases and cancer [7, 28]. Our study participants had overall mild liver disease; thus, the existence of association between hepatic *DPP4* and histological signs of liver damage in individuals with more advanced liver impairment, cannot be definitively ruled out.

Major strength of this study is the coexistence of data on plasma and tissue DPP4 in a large population of metabolically characterized individuals with different grade of obesity, metabolic impairment and NAFLD/NASH. Moreover, the primary study endpoint was set on a cohort of individuals undergoing liver biopsy, which represents to date the gold standard for NAFLD and NASH diagnosis.

This is the first study evaluating contextually both DPP4 concentration and activity, as well as the *DPP4* mRNA expression in liver samples, in relation to the presence of biopsy-proven NAFLD/NASH. Another novel aspect of this study is the replication of the experiments in two parallel cohorts of individuals with different metabolic characteristics, i.e. morbid obese vs. normal-weight individuals, with and without NAFLD and metabolic diseases. Finally, our study is the first one which investigated DPP4 activity and concentration in relation to the FIB-4 score, a broadly validated marker of liver fibrosis which play a central role in the diagnostic algorithm of NAFLD and NASH in high risk populations [36].

A limitation of this study is the cross-sectional design which does not allow to establish a causal nexus between increased plasma DPP4 activity/concentration and NAFLD; longitudinal studies are warranted in order to test the role of DPP4 as a marker of NAFLD development and progression in individuals at high risk, as those with T2DM and metabolic disease.

In conclusion, this study demonstrates the coexistence of increased plasma DPP4 concentration and DPP4 enzymatic activity in patients with NAFLD, with and without obesity and metabolic disorders. Circulating DPP4 measurement may represent a novel cost-effective strategy for NAFLD/NASH risk stratification in dysmetabolic populations and a potential tool for monitoring disease’s progression and response to treatment in established NAFLD.
